# Genomic epidemiology and characterisation of penicillin-sensitive *Staphylococcus aureus* isolates from invasive bloodstream infections in China: an increasing prevalence and higher diversity in genetic typing be revealed

**DOI:** 10.1080/22221751.2022.2027218

**Published:** 2022-01-21

**Authors:** Ye Jin, Wangxiao Zhou, Qing Zhan, Yunbo Chen, Qixia Luo, Ping Shen, Yonghong Xiao

**Affiliations:** a State Key Laboratory for Diagnosis and Treatment of Infectious Diseases, National Clinical Research Center for Infectious Diseases, Collaborative Innovation Center for Diagnosis and Treatment of Infectious Diseases, The First Affiliated Hospital, Zhejiang University School of Medicine, Hangzhou, People’s Republic of China; b Infection Control Department, The First Affiliated Hospital, Zhejiang University School of Medicine, Hangzhou, People’s Republic of China; c Jinan Microecological Biomedicine Shandong Laboratory, Jinan, People’s Republic of China

**Keywords:** Penicillin sensitivity, methicillin-susceptible *Staphylococcus aureus*, *blaZ*, bloodstream infections, genomic epidemiology

## Abstract

Many countries have reported increasing rates of penicillin-susceptible methicillin-sensitive *Staphylococcus aureus* (MSSA-PEN^S^). To date, there is relatively little known about the current situation and molecular characteristics of MSSA-PEN^S^ in China. In this study, we carried out a laboratory-based multi-region retrospective study to investigate the genomic epidemiology and characterisation of MSSA-PEN^S^ isolated from invasive bloodstream infections (BSIs) across 17 provinces. The prevalence of MSSA-PEN^S^ isolates increased significantly over the 6-year period, with the proportion increasing from 3.51% in 2014–8.80% in 2019, an average relative increase of 22.14% per year (95% confidence interval 9.67%-34.61%, P for trend <0.001), suggesting that China is experiencing a resurgence of MSSA-PEN^S^. Phylogenetic analysis showed a higher strain diversity occurred; the most frequent clonal complexes (CCs) identified were CC188 (17.14%), CC398 (15.71%) and CC5 (15.71%). Over half of MSSA-PEN^S^ strains were pan-susceptible, with erythromycin the most frequent resistance observed. Moreover, 25 isolates were identified as immune evasion cluster negative, including CC15, CC188 and CC1, and 6 strains encoded the Panton-Valentine leucocidin gene. Importantly, virulence assays showed that MSSA-PEN^S^ exhibited a level of virulence comparable to that of penicillin-resistant MSSA (MSSA-PEN^R^), indicating that more-sensitive strains should not be mistaken for lacking aggressiveness in *vivo*. Furthermore, 11 of these isolates were confirmed as *blaZ* positive but phenotype sensitive, with different amino acid changes in *blaZ*. Our data support the recommendation to clinicians regarding the usage of penicillin in invasive BSIs caused by MSSA-PEN^S^, which might create a novel opportunity for better antimicrobial stewardship in the future.

## Introduction

*Staphylococcus aureus* is an opportunistic pathogen with high capacity to adapt to human hosts and healthcare settings. With the rise of hospital-based medicine, *S. aureus* quickly became a major cause of health-care-associated infections especially bloodstream infections (BSIs) [1,2], with two-thirds of cases being attributed to methicillin-sensitive *S. aureus* (MSSA). Patients with MSSA BSIs can develop complications, leading to poor clinical outcomes [1,3], and mortality rates associated with MSSA bacteraemia range from 9% to 50% [4–7].

The emergence of penicillin in the 1940s offered short-lived relief regarding *S. aureus* infections [8]. However, with the widespread use of penicillin, penicillin resistance among *S. aureus* isolates arose within several years, mediated by the β-lactamase gene *blaZ* [9]. As a result, penicillinase-stable β-lactams such as cefazolin and cloxacillin have taken the place of penicillin in MSSA treatment. Given the availability of these β-lactams and the very high rates of penicillin resistance, penicillin susceptibility testing is rarely performed in the laboratory. Nevertheless, in recent years, researchers have found that the rate of BSIs caused by penicillin-susceptible MSSA (MSSA-PEN^S^) is gradually increasing in many countries [10–15], indicating that penicillin susceptibility may be in a period of renaissance.

China is a developing country with high rates of antibiotic prescriptions on their use. Therefore, to better understand the current status of MSSA-PEN^S^ in China for the purpose of facilitating effective additions to treatment options for MSSA BSIs, in this study, we investigated the prevalence and molecular features of MSSA-PEN^S^ in BSIs as well as the characterisation of these MSSA-PEN^S^ isolates over a time span of 6 years in 55 hospitals across 37 cities in 17 provinces.

## Materials and methods

### Study design and S. aureus isolates collection

As this project was a laboratory-based, multi-region retrospective study of *S. aureus* permitted by the Research Ethics Board of the First Affiliated Hospital of Zhejiang University School of Medicine, the requirement for informed consent was waived, and all patients who volunteered to participate in this study at 55 hospitals in 37 cities across 17 provinces of China were anonymised. A total of 2701 non-repetitive *S*. *aureus* strains (1952 MSSA and 749 MRSA isolates) were isolated from blood cultures of patients with BSIs between 2014 and 2019.

### Antimicrobial susceptibility determination

Antibiograms of isolates to 20 antimicrobial agents (oxacillin, penicillin G, ciprofloxacin, levofloxacin, moxifloxacin, sulfamethoxazole and trimethoprim, amikacin, gentamicin, clindamycin, erythromycin, rifampin, tetracycline, tigecycline, fusidic acid, mupirocin, vancomycin, linezolid, teicoplanin and daptomycin) were prepared using the agar dilution method following the Clinical and Laboratory Standards Institute guideline. The designation of *S. aureus* isolates as MSSA was based on oxacillin susceptibility (minimal inhibitory concentration [MIC] ≤2 mg/L). The penicillin susceptibility of MSSA isolates was confirmed by disc diffusion (1-U disc).

### Whole-genome sequencing (WGS) and genomic analysis of MSSA-PEN^S^ isolates

Genomic DNA was extracted from 140 MSSA-PEN^S^ isolates using an Ezup Column Bacteria Genomic DNA purification kit (Sangon Biotech, Shanghai, China) and sequenced on a HiSeq X Ten platform (Illumina, San Diego, CA, USA). The 150-bp paired-end reads were generated and then processed for quality filtering and adaptor trimming using fastp v0.20.1 [16]. The clean data were then assembled using SPAdes v3.14.1 [17]. SpaFinder (https://cge.cbs.dtu.dk/services/spatyper) was used to confirm the *spa* typing of MSSA-PEN^S^ isolates. Antimicrobial resistance genes and virulence factors were detected using ResFinder v4.0 [18] and VFanalyzer (http://www.mgc.ac.cn/cgi-bin/VFs/v5/main.cgi?func=VFanalyzer), respectively. Antimicrobial resistance associated with chromosomal mutations was identified using PointFinder v3.2 [19]. Type of the *blaZ* gene (A–D) was determined based on the amino acids at positions 119 and 207 of the gene product [20]. Mutations in the *blaZ*, *blaI*, and *blaR1* genes of MSSA-PEN^S^-*blaZ*^positive^ isolates were identified using the sequence of *S. aureus* ATCC 29213 as a reference. We used Roary v3.13.0 [21] to perform pan-genome analyses on all 140 MSSA-PEN^S^ isolates; a phylogenetic tree was constructed based on the single-nucleotide polymorphism (SNP) alignment of the core-genome alignment (from Roary output) using RAxML v8.2.11 [22]. The resulting tree was rooted at the midpoint and visualised using the ggtree R package [23].

### Comparison of virulence between MSSA-PEN^S^ and MSSA-PEN^R^ isolates

Virulence assays were performed as previously described[24]. Strains were incubated in TSB at 37°C. Mouse skin infection and *Galleria mellonella* larvae infection models were constructed for virulence determination. Briefly, each larva (n = 30) was injected with 1×10^5^ cells, and the number of dead larvae was recorded within 48 h. Each mouse (n = 5) was injected with 1×10^6^ cells suspended in 100 μL of NaCl solution. Skin lesions were measured (length [L] × width [W]) for 4 days.

## Results

### Prevalence of penicillin susceptibility of MSSA was increased in China

**Figure 1.**
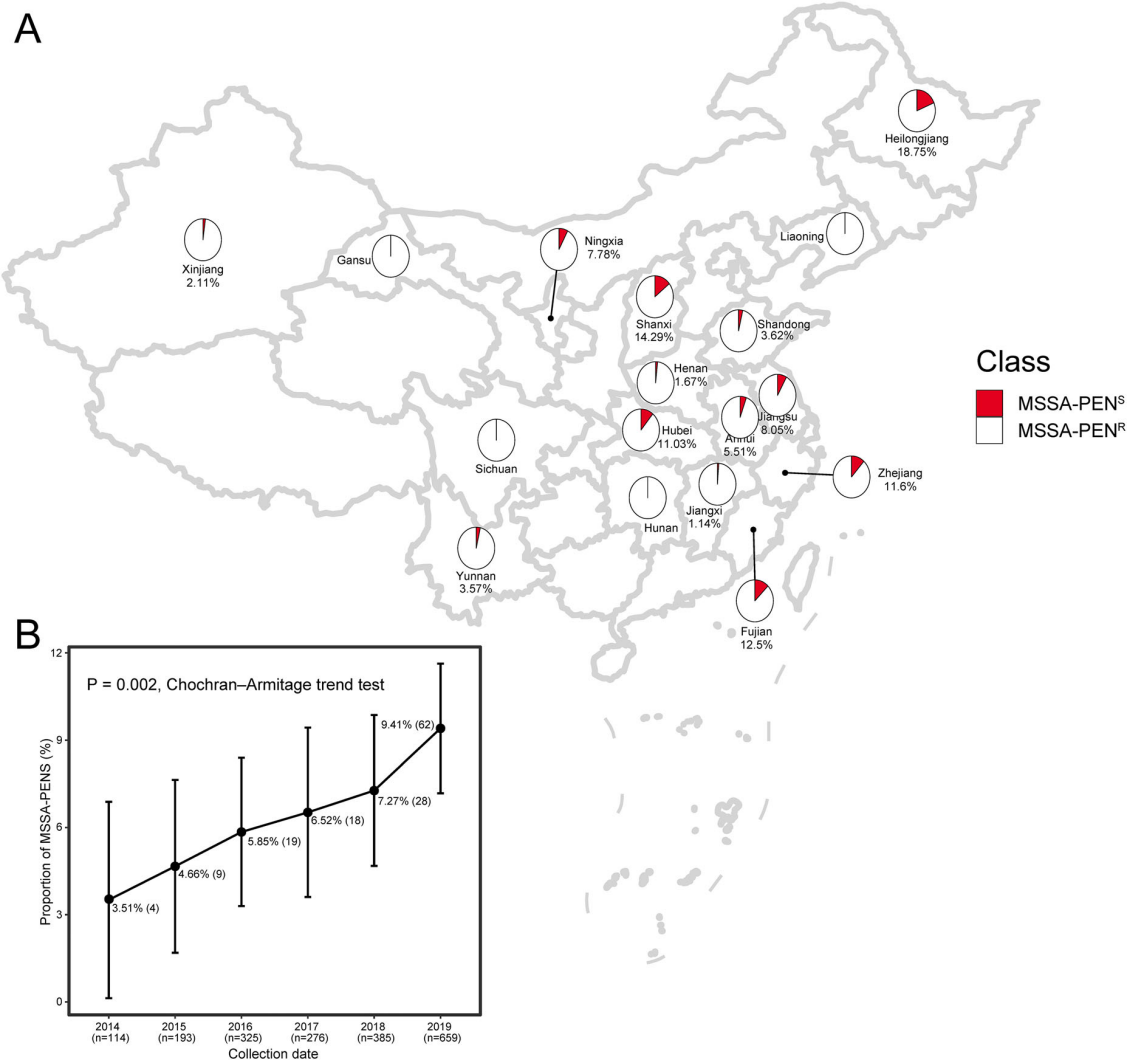
Distribution and proportion of MSSA-PEN^S^ isolates in this study. (A) Geographical distribution of 1952 MSSA isolates across 17 provinces in China. Red and white dots represent MSSA-PEN^S^ and MSSA-PEN^R^, respectively. (B) Proportion of MSSA-PEN^S^ isolates by year among all MSSA isolates. The increasing trend was analysed statistically using the Cochran-Armitage trend test.

A total of 1952 MSSA strains isolated from blood were examined in this study. As shown in Figure 1A, among 17 provinces, strains isolated from Gansu, Sichuan, Hunan and Liaoning provinces were all resistant to penicillin. The region with the highest rate of penicillin sensitivity was Heilongjiang, with a rate of 18.75% (3/16, 18.75%). Shanxi Province had the second highest rate, at 14.29% penicillin sensitivity among MSSA strains (2/14, 14.29%). Rates of penicillin sensitivity among MSSA in five provinces [Heilongjiang (3/16,18.75%), Shanxi (2/14, 14.29%), Fujian (18/144, 12.5%), Zhejiang (50/431, 11.6%) and Hubei Province (15/136, 11.0%)] were >10%.

The proportion of MSSA-PEN^S^ detected among all MSSA isolates from blood cultures identified in the 55 Chinese hospitals was 7.17% (140/1952). Of note, as shown in Figure 1B, we found that the prevalence of MSSA-PEN^S^ increased significantly among the MSSA isolates over the 6-year period, with the proportion increasing from 3.51% in 2014–8.80% in 2019, an average relative increase of 22.14% per year (95% confidence interval [CI] 9.67%-34.61%, P for trend <0.001).

### Genome sequencing–based determination of the population structure of MSSA-PEN^S^ isolates from BSIs in China

**Figure 2.**
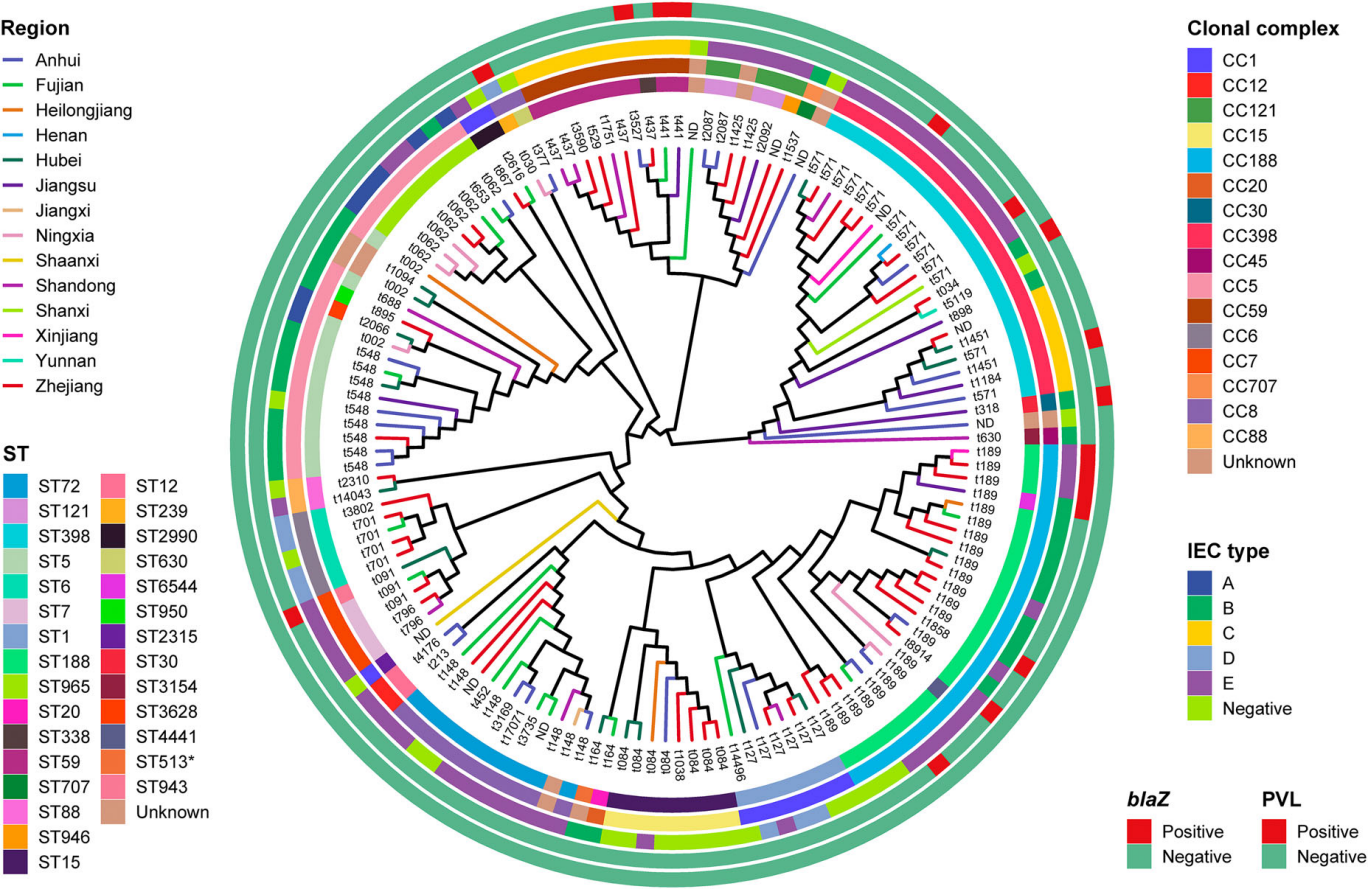
Phylogenetic tree of 140 MSSA-PEN^S^ isolates. Different colours of branches in the tree represent different provinces from which isolates were collected. Data on the CC types, ST types, IEC types and presence of *blaZ* gene and PVL are mapped on the tree from inner to outer circle. Tips are labelled according to *spa* types of isolates.

To accurately determine the population structure of MSSA-PEN^S^ isolates from BSIs in patients at 55 hospitals across China, we first undertook a whole-genome phylogenetic analysis. As shown in Figure 2, among 140 MSSA-PEN^S^ isolates, 26 STs were identified, and seven isolates were defined as new singletons. The 26 identified ST types belong to 16 clonal complexes (CCs), with CC188 (17.14%), CC5 (15.71%), CC398 (15.71%), CC8 (9.29%) and CC1 (7.14%) the most frequently detected CCs, comprising >65.0% of all MSSA-PEN^S^ isolates. Of note, a higher strain diversity was observed yearly from 2014 to 2019 (Figure S1). We observed that >40% of MSSA-PEN^S^ isolates belonged to sporadically occurring clones, such as ST707, ST2315, ST1821 and ST2990.

Of the isolates, 11 strains displaying penicillin sensitivity but harbouring *blaZ* were observed in this study. Thus, we defined MSSA-PEN^S^ harbouring *blaZ* as MSSA-PEN^S^-*blaZ*^positive^(n = 11) and MSSA-PEN^S^ that did not harbour *blaZ* as MSSA-PEN^S^-*blaZ*^negative^(n = 129). Among the 11 MSSA-PEN^S^-*blaZ*^positive^ isolates, most belonged to CC188 (63.64%, 7/11), whereas the remaining isolates were CC398 (n = 2), CC7 (n = 1) and CC8 (n = 1). Furthermore, most MSSA-PEN^S^-*blaZ*^positive^ strains (6/11) were isolated from Zhejiang Province.

Spa-typing further refined the population structure of these isolates. We observed 88 distinct spa types in all, with t189, t571, and t548 being the most prevalent. Furthermore, substantial correlations among spa types and CCs were identified in this study (Figure 2). t189 was the predominant *spa* type, constituting 15.71% of all strains. All isolates of the t189 type belonged to CC188. MSSA-PEN^S^ strains of the second most common *spa* type, t571, accounted for 10.0% of the isolated strains, 63.64% of which were CC398. Notably, t571 is the *spa*-type most associated with CC398-MSSA BSIs in Europe [25,26]. Furthermore, isolates of t548 accounted for 6.98% of MSSA-PEN^S^ strains, and all belonged to CC5. Notably, nine isolates of novel *spa* type were detected among all 129 MSSA-PEN^S^ strains.

### Analysis of resistance phenotype and genotype and virulence genes among MSSA-PEN^S^-blaz^negative^ isolates

**Figure 3.**
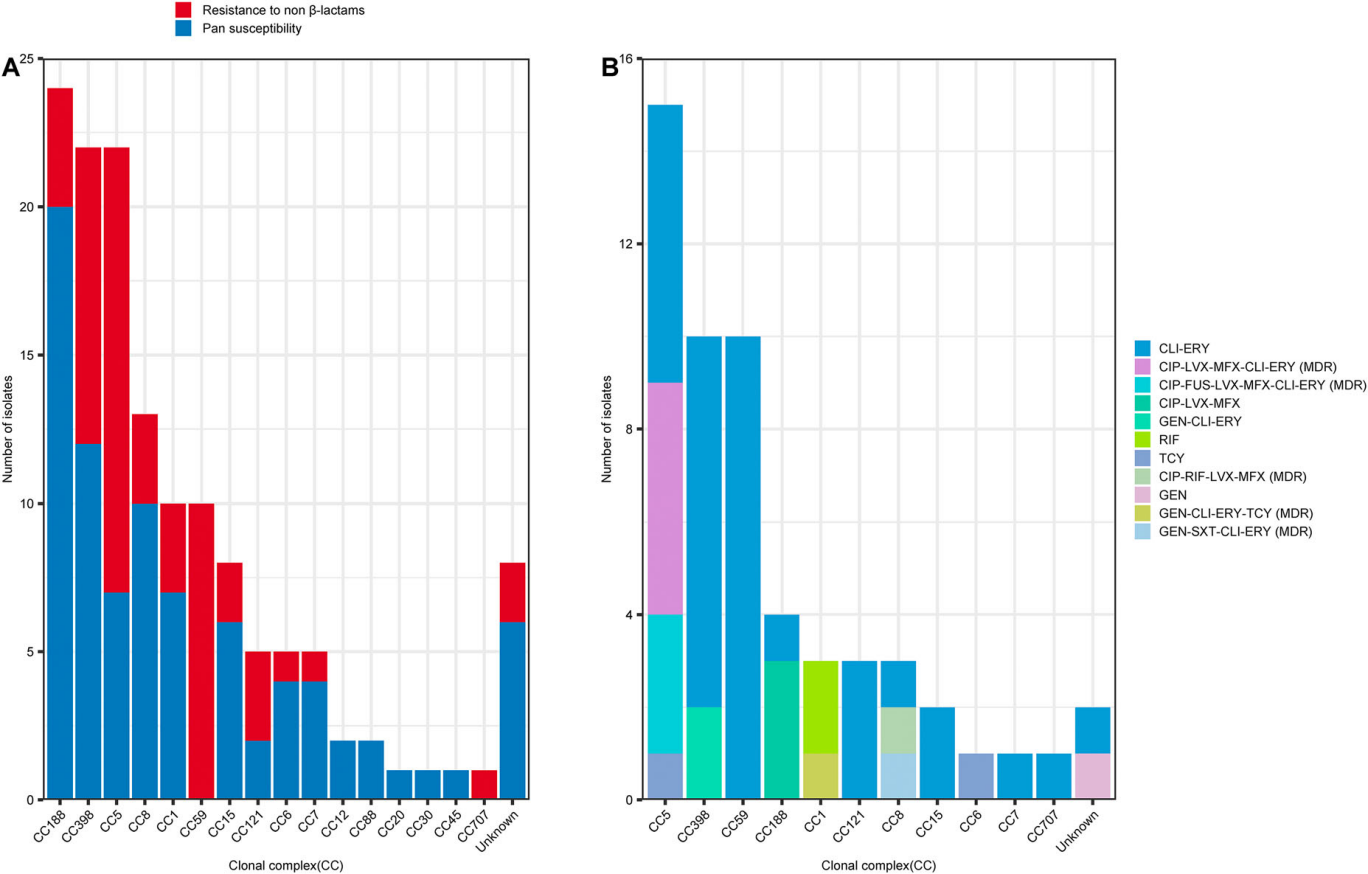
Relationship between CCs and antimicrobial resistance phenotype detected among the 129 MSSA-PENS-*blaZ*^negative^ isolates. (A) Pan-susceptible strains or isolates resistant to non-β-lactams detected among the different CCs. (B) CCs detected among isolates showing resistance to non-β-lactams and the antimicrobial resistance phenotype identified in those isolates. CLI-ERY, clindamycin-erythromycin resistance; CIP-LVX-MFX-CLI-ERY, ciprofloxacin-levofloxacin-moxifloxacin-clindamycin-erythromycin resistance; CIP-FUS-LVX-MFX-CLI-ERY, ciprofloxacin fusidic acid-levofloxacin-moxifloxacin-clindamycin-erythromycin resistance; CIP-LVX-MFX, ciprofloxacin-levofloxacin-moxifloxacin resistance; GEN-CLI-ERY, gentamicin-clindamycin-erythromycin resistance; RIF, rifampin resistance; TCY, tetracycline resistance; CIP-RIF-LVX-MFX, ciprofloxacin-rifampin-levofloxacin-moxifloxacin resistance; GEN, gentamicin resistance; GEN-CLI-ERY-TCY, gentamicin-clindamycin-erythromycin-tetracycline resistance; GEN-SXT-CLI-ERY, gentamicin-sulfamethoxazole/trimethoprim-clindamycin-erythromycin resistance; MDR, multi-drug resistance.

As shown Table S1, most of the MSSA-PEN^S^-*blaZ*^negative^ strains (58.14%, 75/129) exhibited pan-susceptibility (susceptible to all 20 antimicrobials tested). No CC59 and CC707 isolates displayed pan-susceptibility (Figure 3A). In the remaining strains, resistance to erythromycin (34.9%, 45/129), clindamycin (18.6%, 24/129), ciprofloxacin (11/129, 8.53%), levofloxacin (12/129, 9.30%), moxifloxacin (11/129, 8.53%), tetracycline (3.10%, 4/129), gentamicin (2.33%, 3/129), and fusidic acid (2.33%, 3/129) was detected. One sulfamethoxazole/trimethoprim (0.78%, 1/129) resistant strain was isolated and belonged to CC8. As shown in Figure 3A, except CC12, CC88, CC20, CC30 and CC45, non-β-lactam resistance was found in isolates belonging to CC188, CC398, CC59, CC5 clones et.al, although most of the resistant strains were CC398, CC5 and CC8. Only 12 isolates were multi-drug resistant (MDR, resistant to at least three antimicrobial families), and most of the MDR strains belonged to CC5 (76.92%, 10/13) and CC8 (3/3, 100%). As the dominant CC of hospital-acquired infections (in both MSSA and MRSA infections), CC5 and CC8 exhibited more resistance to antibiotics than did the other *S. aureus* clones. Furthermore, among the resistant strains, as shown in Figure 3B, most (34/55, 61.8%) were only resistant to clindamycin and erythromycin (CLI-ERY), including CC59(n = 10), CC59 (n = 10, CC398 (n = 8), CC5 (n = 6) and CC121 (n = 3) et.al. Rifampin resistance only occurred in CC1 isolates.

**Figure 4.**
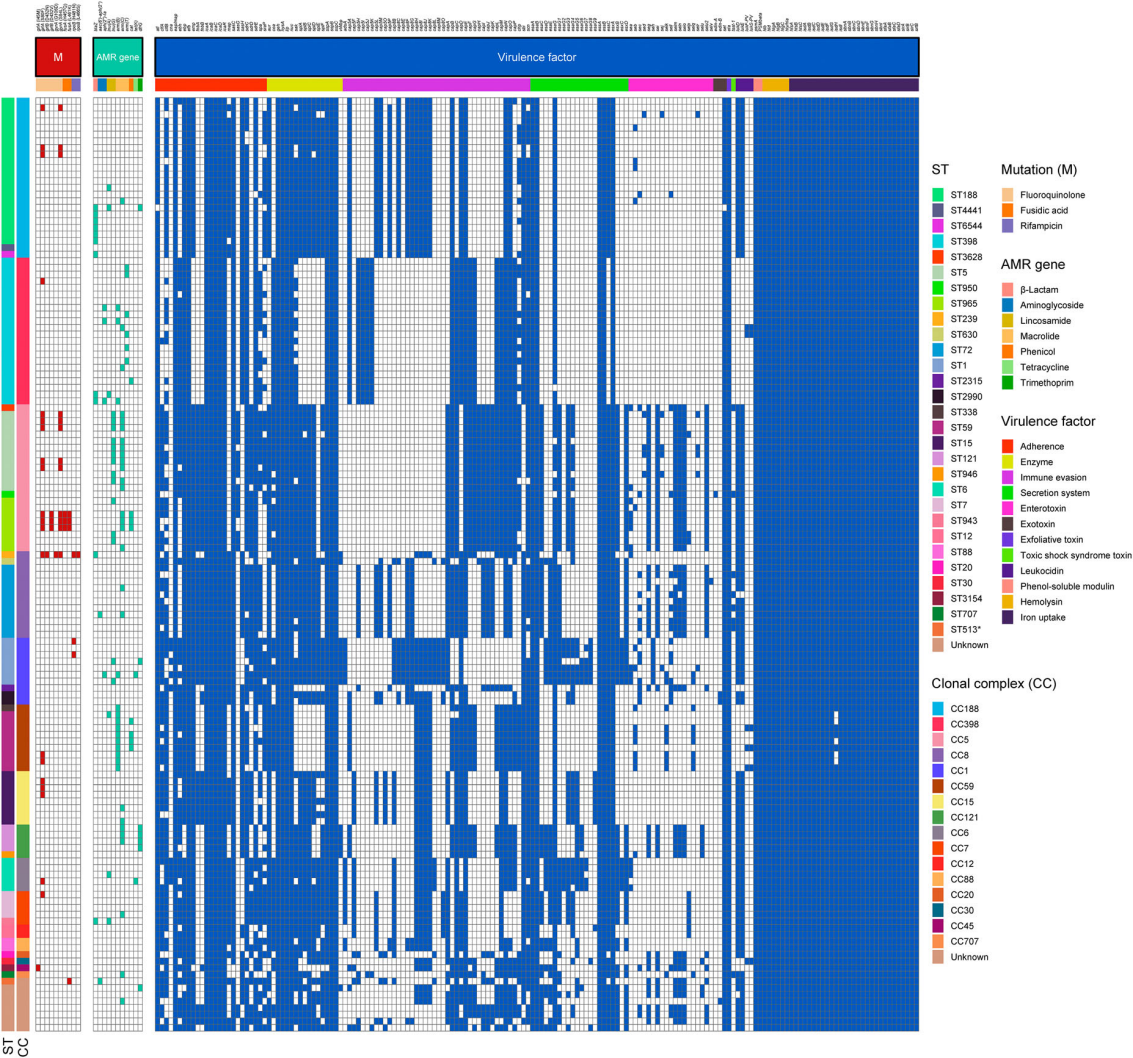
Heatmap of antibiotic resistance and virulence genes among the 140 MSSA-PEN^S^ isolates.

A total of nine resistance genes associated with macrolides (*ermB*, *ermC*, *ermT*), lincosamides (*lnu[A]*, *lnu[G]*), aminoglycosides (*aac[6′]-aph[2″]*, *aph[2″]-Ia)*, phenicol (*cat*), trimethoprim (*drfG*) and tetracycline (*tetK*) were detected in MSSA-PEN^S^ isolates (Figure 4). Mutations in *grlA*, *gyrA*, *fusA* and *rpoB* were also identified among these strains. Consistent with the results of the predicted genotypes, as shown in Figure 4, mutations in *fusA* (H457Q and L461 K) were present in three fusidic acid–resistant CC5 (ST965) clones, whereas the rifampicin resistance *rpoB* H481N and *rpoB* L466S mutations were only detected in rifampicin-resistant CC8 (ST239) and CC1 (ST1) clones. Furthermore, six MSSA-PEN^S^ isolates that had an inducible macrolide-lincosamide-streptogramin B resistance phenotype, which is mediated by *ermT*, were all CC398 (Figure 4). Notably, these six CC398 isolates were all resistant to erythromycin (ERY) and clindamycin (CLI), and all belonged to an ancestral human-adapted clade with the integrase group 3 prophage (φSa3) containing the immune evasion cluster (IEC) genes and the erythromycin-resistance gene *ermT*.

**Figure 5.**
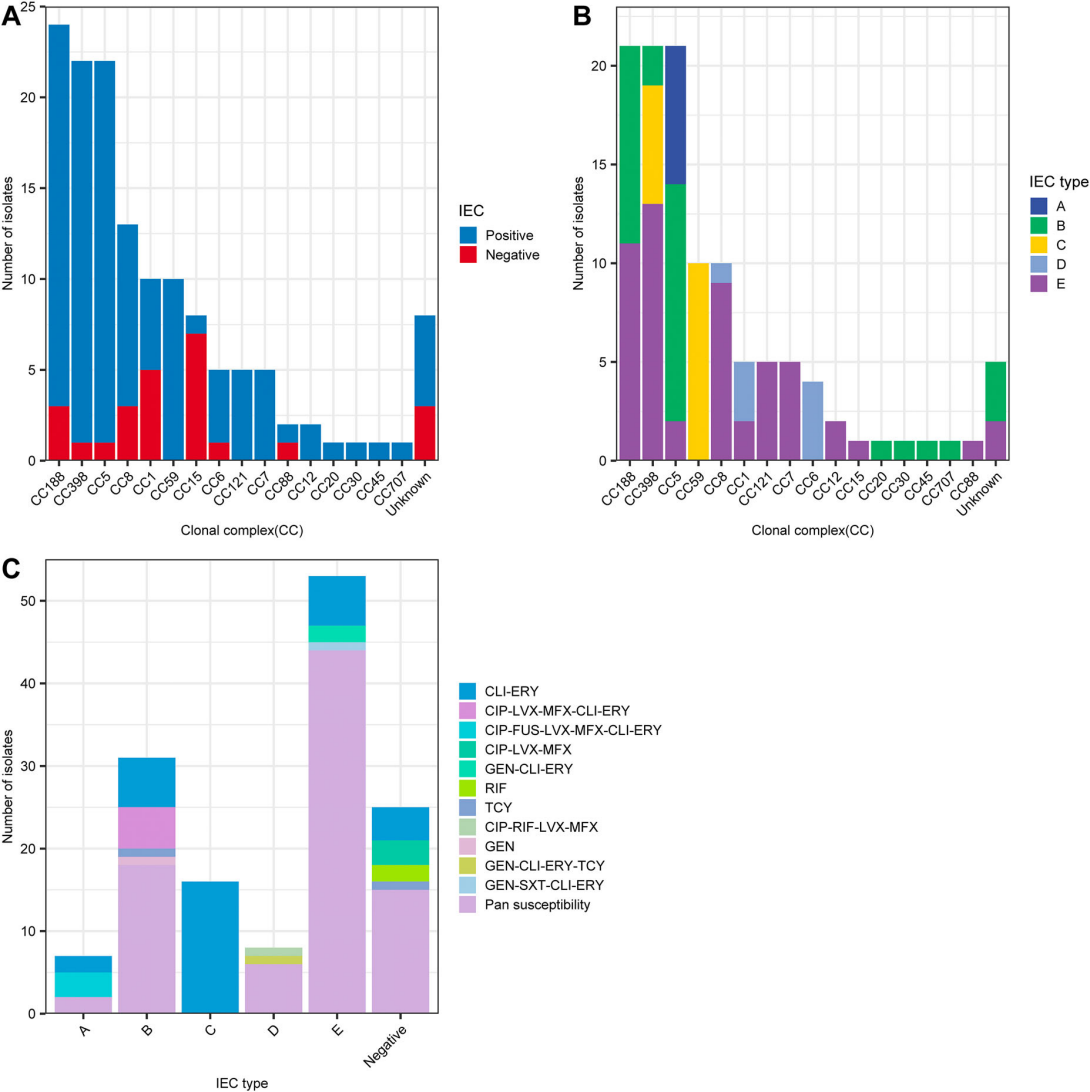
Relationship between CCs, IEC, and antimicrobial resistance phenotypes among 129 MSSA-PEN^S^-*blaZ*^negative^ strains. (A) Distribution of IEC among different CCs identified in 129 MSSA-PEN^S^ isolates. (B) Distribution of different IEC types among different CCs. (C) Different antimicrobial resistance phenotypes among different IEC types among 129 MSSA-PEN^S^-*blaZ*^negative^ strains.

The IEC is a genetic element that encodes chemotaxis inhibitory protein (*chp*), staphylococcal complement inhibitor (*scn*), chemotaxis inhibitory protein (*chp*), staphylokinase (*sak*), staphylococcal enterotoxin A (*sea*) and staphylococcal enterotoxin P (*sep*). These human evasion genes have a specific interaction on components of the human innate immune system [7]. In this study, 25 isolates were identified as IEC negative, including CC15, CC188 and CC1, among others (Figure 4). It is important to emphasize that we observed one IEC-negative strain (CC398), suggesting that this strain originated from an animal source. As shown in Figure 5A, all strains that belonged to clonal lineages CC59, CC121, CC7, CC12, CC20, CC30, CC45 and CC707 were IEC positive. Moreover, the remaining 115 IEC-positive strains were divided into five types according to analysis of the five genes of the IEC system: A (*sea*-*sak*-*chp*-*scn*, n = 7), B (*sak*-*chp*-*scn*, n = 31), C (*chp*- *scn*, n = 16), D (*sea-sak-scn,* n = 8) and E (*sak-scn*, n = 53). As shown in Figure 5B, type E constituted the major proportion of MSSA-PEN^S^-*blaZ*^negative^ isolates, with CC398 the most frequent CC (13/53), followed by CC188 (11/53) and CC8 (9/53). In type E, the *chp* gene was missing. The second most common IEC type was type B, which consisted of CC188, CC398 and CC5.eg. In addition, all CC59 and six of the CC398 isolates belonged to type C. In our previous work, we found that almost all ST59-MRSA V clones and all ST398-MRSA clones lacked the *sak* gene and belonged to IEC type C, whereas ST59-MRSA-IV clones were type B [27]. As shown in Figure 5C, among these IEC-positive strains, resistance was detected in isolates of IEC types A–E. Some resistance phenotypes, such as rifampin and (CIP-LVX-MFX) resistance, were only identified in IEC-negative strains. Pan-susceptibility strains were found in IEC types ABDE that were IEC negative, whereas IEC type C strains were only resistant to CLI-ERY.

Panton-Valentine leucocidin (PVL) genes (*lukS/F*) were detected in six MSSA-PEN^S^-*blaZ*^negative^ isolates (6/140, 4.29%) belonging to CC59, CC398 and CC30 (Figure 4). It is not uncommon for MSSA or MRSA strains belonging to CC59 and CC30 and CC398 MSSA isolates to harbour *lukS/F* gene [27–29]; however, in general, *lukS/F* gene carriage is rare in livestock-associated (LA)-MRSA CC398. In this study, two CC398 strains (ST398-t034-IEC-B and ST398-t1451-IEC-C) were found to harbour *lukS/F* gene, and both were tetracycline sensitive.

### Characterization of MSSA-PEN^S^-blaz^positive^ isolates

Eleven MSSA-PEN^S^ isolates were *blaZ*^positive^. Detailed information regarding these strains is provided in Table 1. Repeated penicillin susceptibility tests were carried out to confirm phenotypically that these strains were susceptible to penicillin. Almost all MSSA-PEN^S^-*blaZ*^positive^ strains (10/11) harboured the type C *blaZ* gene, whereas the remaining strain harboured type A. Most of these MSSA-PEN^S^-*blaZ*^positive^ isolates were pan-sensitive, except one ST239-t030 strain, which belonged to both *blaZ* type A and IEC type D and was resistant to rifampin, ciprofloxacin, moxifloxacin and levofloxacin. As one of the dominant hospital-acquired MRSA clones in Asia [30,31], ST239 has persisted in healthcare settings for decades, and the clone has adaptively evolved and become MDR.

**Table 1.** Characteristics of 11 MSSA-PEN^S^- *blaZ* ^positive^ isolates collected from China

Strain	ST	CC-*spa* type	Resistance phenotype	Penicillin MIC (mg/L)	Disc diffusion, 1 U penicillin disc (diameter mm)	IEC type	*blaZ* type
SKLX44218	ST398	CC398-t571	Susceptible	0.03	32	E	C
SKLX56709	ST188	CC188-t189	Susceptible	0.06	30	E	C
SKLX58097	ST188	CC188-t189	Susceptible	0.03	40	E	C
SKLX61252	ST943	CC7-t091	Susceptible	0.03	30	E	C
SKLX62838	ST239	CC8-t030	RIF, CIP, LVX, MFX^a^	0.06	35	D	A
SKLX36700	ST188	CC188-t189	Susceptible	0.03	39	B	C
SKLX56830	ST188	CC188-t189	Susceptible	0.03	41	E	C
SKLX56858	ST188	CC188-t189	Susceptible	0.03	40	E	C
SKLX58839	ST398	CC398-t571	Susceptible	0.03	42	E	C
SKLX60497	ST188	CC188-t8914	Susceptible	0.03	39	E	C
SKLX63241	ST6544	CC188-t189	Susceptible	0.03	40	B	C

^a^ RIF, rifampicin; CIP, ciprofloxacin; LVX, levofloxacin; MFX, moxifloxacin.

Several additional amino acid changes were detected among these isolates (Figure S2). For instance, the mutations in isolates SKLX44218, SKLX56709 and SKLX61252 introduced a frameshift (c.92delA) introducing a premature stop codon at codon 43 (L43*); the G223 T substitution in isolate SKLX62838 resulted in a premature stop codon at codon 75 (E75*); and the nonsynonymous amino acid substitutions occurring in the other isolates comprised those previously reported in the CC5-t002 isolate X1953 [32]. We also examined the mutations in the *blaI* gene for these 11 isolates and found that there were no changes in the sequences of the *blaI* gene in isolates SKLX56709 and SKLX61252, whereas isolate SKLX62838 exhibited a change in codon 21 (p.D21G), and the other isolates showed changes in codon 2 (p.A2 T). However, isolate SKLX62838 did not exhibit amino acid changes in the deduced sequence of BlaR1, whereas the isolates SKLX44218, SKLX56709 and SKLX61252 showed 28 amino acid substitutions in *blaR1* gene product, and the same frameshift mutations observed in the other isolates were introduced (c.466delA), introducing a premature stop codon at codon 160 (V160*) (Figure S3). Of note, isolates SKLX56709, SKLX61252, SKLX58097, SKLX36700, SKLX56830, SKLX56858, SKLX58839, SKLX60497 and SKLX63241 shared an identical *bla* operon sequence (*blaZ*-*blaR1*-*blaI*). Isolates SKLX58097 (from Xinjiang, China), SKLX56830 (from Zhejiang, China) and SKLX56858 (from Zhejiang, China) shared no SNPs in the core genome, suggesting potential interregional dissemination of MSSA-PEN^S^-*blaZ*^positive^ isolates in China.

### MSSA-PEN^S^ isolates exhibited a level of virulence comparable to that of MSSA-PEN^R^ isolates

**Figure 6.**
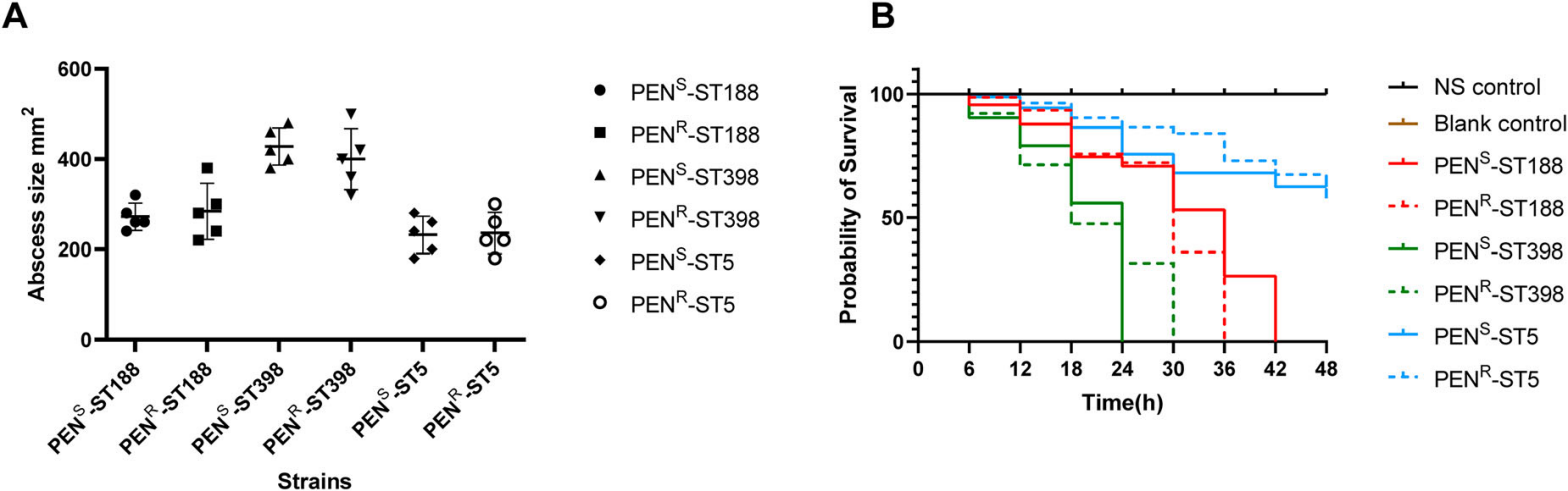
Comparative virulence levels of MSSA-PEN^S^ and MSSA-PEN^R^ strains. (A) Skin lesions caused by MSSA-PEN^S^ and MSSA-PEN^R^ strains in a mouse skin infection model. (B) Survival rates of *Galleria mellonella* larvae exposed to MSSA-PEN^S^ and MSSA-PEN^R^ isolates.

To investigate whether more-sensitive strains showed attenuated virulence, we next investigated the resistance phenotype, genotype and virulence gene analysis between MSSA-PEN^R^ and MSSA-PEN^S^ isolates (CC188, CC398 and CC5). As shown in Figure S4, we found that there was no significant difference in the virulence genes between MSSA-PEN^S^ and MSSA-PEN^R^ strains with the same pattern of antimicrobials sensitivity, antimicrobial genes and ST-*spa* types. Then we compared the virulence of MSSA-PEN^S^ and MSSA-PEN^R^ strains *in vivo*. Strains were chosen to have the same pattern of antimicrobials sensitivity and ST-*spa* types except a difference in resistance to penicillin. As shown in Figure 6A, the area of skin abscesses in mice caused by MSSA-PEN^S^ isolates was similar in size to that of mice infected with MSSA-PEN^R^ isolates (*P*>0.05), suggesting that MSSA-PEN^S^ isolates are as aggressive as MSSA-PEN^R^ isolates. To further confirm the pathogenicity of MSSA-PEN^S^, a *G. mellonella* infection model was constructed. As shown in Figure 6B, the percentage of surviving larvae exposed to MSSA-PEN^S^ was slightly higher compared with exposure to MSSA-PEN^R^, but the difference was nonsignificant (*P*>0.05), indicating that MSSA-PEN^S^ isolates remained quite virulent.

## Discussion

Before penicillin was discovered by Sir Alexander Fleming, it was possible for an otherwise perfectly heathy individual to die of septicaemia from a casual cut. However, by the mid-1940s, only several years after its introduction into clinical practice, penicillin resistance had emerged and continued to worsen in hospitals over the subsequent decades, causing a great threat to human health. Today, due to the coincident rise of “superbugs” such as MRSA and aggravation of bacterial drug resistance, humanity faces the stark reality of a potential post-antibiotic era.

While scientists have paid increasing attention to more-resistant strains, and studies have often underestimated the significance of methicillin-sensitive *S. aureus* strains for years, penicillin-sensitive *S. aureus* strains appear to have crept back in “under the radar”. In some developed countries, such as the USA, Spain, France and Canada, researchers have reported that the proportion of penicillin-susceptible *S. aureus* is approximately 15–45% [11,15,25,32]. Penicillin is a narrow-spectrum agent that offers several advantages, such as lower average MIC and treatment cost and better theoretical pharmacokinetic and pharmacodynamic profiles compared with the usual first-line treatments. However, due to the very high rates of penicillin resistance among MSSA isolates, penicillin is not commonly used in treatment of infections caused by MSSA in China, especially invasive infections. Therefore, more attention should focus on investigations of penicillin susceptibility, as this could lead to improvements in the clinical application of other first-line options.

Thus, we carried out a laboratory-based, multi-region retrospective study to investigate the genomic epidemiology and characterization of penicillin-sensitive *S. aureus* isolated from patients with invasive BSIs across 17 provinces in China over a 6-year period using WGS. In this study, we found that the prevalence of MSSA-PEN^S^ isolates increased significantly among the 1952 MSSA isolates over the 6-year period, with the proportion increasing from 3.51% in 2014–8.80% in 2019, an average relative increase of 22.14% per year (95% CI 11.52%–32.90%, P for trend <0.001). Among these provinces, the highest rate of penicillin sensitivity was 18.75% in Heilongjiang Province. Although the penicillin sensitivity rate in China is not as high as in other developed countries, it has also shown an upward trend year by year.

The most frequent CCs identified among all MSSA-PEN^S^ strains were CC188 (17.14%), CC398 (15.71%), CC5 (15.71%), CC8 (9.29%) and CC1 (7.14%), which comprised >65.0% of all MSSA-PEN^S^ isolates. Previous studies have reported that CC188 is one of the most common clones causing BSIs and often associated with MSSA, and this clone is more likely to cause hospital-acquired infections in adult patients and community-associated infections in paediatric patients [33–35]. CC398 has emerged as an invasive clone in many countries [36,37], with increasing prevalence reported over the years. CC398 is broadly separated into two clusters: livestock-associated MRSA and human-adapted MSSA [38].

In this study, almost all CC398 isolates belonged to *spa*-type t571, which has been identified as part of a human-adapted CC398 MSSA clade [39]. Furthermore, these CC398 isolates exhibited features of the human-adapted cluster, contained an IEC system, and were sensitive to tetracycline. Only one additional CC398 isolate was found to lack an IEC, indicating a livestock origin. However, this CC398 strain did not show the characteristics of the LA clade (absence of IEC, presence of *tet[M]* gene and absence of *erm[T]* gene), as this strain was sensitive to tetracycline and lacked the *tet(M)* gene. In addition, two CC398 isolates harbouring the PVL gene were found in this study. We identified the two PVL-positive CC398 strains as ST398/t034-carrying/TCY-S/ERY-R/IEC type B and ST398/t1451-carrying/TCY-S/ERY-R/IEC type C, respectively. It is important to emphasize that the human-adapted CC398 MSSA was identified as a predecessor of community-acquired MRSA CC398, which evolved by acquiring SCC*mec* cassettes imparting methicillin resistance [40]. CC5 is one of the most dominant clones among both MRSA and MSSA in China [27]. In addition, CC5 has also been demonstrated as one of the most prevalent clones among MSSA-PEN^S^ strains in other studies [32,41].

Pan-susceptibility was identified in over a half of the MSSA-PEN^S^-*blaZ*^negative^ isolates (58.14%). Erythromycin exhibited the poorest antibacterial activity (resistance rate of 32.86%), with most of the erythromycin-resistant isolates belonging to CC5, CC398 and CC59, and the resistance was encoded by the *ermB*, *ermC* and *ermT* genes, as predicted by WGS. Notably, although most of the MSSA-PEN^S^ isolates identified in this study were *blaZ*^negative^ (92.14%, 129/140), WGS also confirmed that 11 isolates were *blaZ*^positive^ with different amino acid changes. As a first-generation cephalosporin, cefazolin is the priority antibiotic that is routinely available to treat invasive MSSA infections [42]. Cefazolin inoculum effect (CzIE) is a phenomenon mediated by staphylococcal β-lactamases[20], which has been demonstrated to be associated with therapeutic failures and increased mortality[43]. Interestingly, we found that in addition to some nonsynonymous amino acid substitutions occurring in the *blaZ* gene reported early [32], a single-nucleotide deletion (c.92delA) was detected in three isolates, introducing a frameshift and premature stop codon predicted to induce expression of truncated *blaZ*. Similarly, we also found a single-nucleotide deletion within the *blaR1* gene (c.466delA) in seven isolates that introduced a premature stop codon and suppressed *blaR1* expression. Previous study has investigated the effect of different *blaZ* types and polymorphisms at specific amino acid positions of BlaZ on the CzIE [20]. In addition, 92delA and 466delA were identified as mutation hot spots for the *blaZ* and *blaR1* genes, respectively. This finding indicates that single-nucleotide deletions might play a significant role in penicillin susceptibility of MSSA. However, the role of nonsynonymous amino acid substitutions in penicillin susceptibility should be investigated further in the future.

We also investigated whether higher antibiotic sensitivity was associated with less aggressiveness *in vivo*. For this purpose, we compared the virulence of MSSA-PEN^S^ and MSSA-PEN^R^ isolates. Interestingly, MSSA-PEN^S^ isolates did not exhibit attenuated virulence. Due to its antimicrobial sensitivity, infections caused by MSSA-PEN^S^ isolates are easier to treat than those caused by more-resistant strains. However, based on our study, we propose that the danger posed by MSSA-PEN^S^ isolates should not be underestimated, as they can cause infections as severe as those caused by MSSA-PEN^R^ isolates.

In conclusion, like other developed countries such as Sweden, the USA and Spain, China has also experienced a resurgence of penicillin susceptibility among MSSA strains. In this study, we confirmed increased penicillin susceptibility among MSSA isolates obtained from patients with invasive BSIs. Although variable, in some provinces, more than 10% the MSSA isolates were susceptible to this conventional agent. To date, the reasons for the resurgence of penicillin susceptibility remain unknown. Long-term empiric therapy of *S. aureus* infections using broad-spectrum antimicrobials might have led to penicillin becoming a “forgotten” antimicrobial in clinical treatment, and the heavy reliance upon vancomycin has also provided opportunities for the emergence of these sensitive isolates. Alternatively, the emergence of novel successful penicillin-susceptible *S. aureus* clones may provide another possible explanation for the increasing rate of MSSA-PEN^S^. Continuous investigations should be performed to clarify whether some successful penicillin-susceptible *S. aureus* clones have emerged, which may be responsible for the increasing rates of MSSA-PEN^S^. Taken together, our integrated epidemiologic analysis and characterisation of the MSSA-PEN^S^ isolates indicated that penicillin susceptibility is worthy of research attention and that more-sensitive strains should not be mistakenly considered to lack aggressiveness *in vivo*. Considering the progressive increase in MSSA in BSIs, our data support the recommendation to clinicians regarding the usage of penicillin in invasive BSIs caused by MSSA-PEN^S^ isolates, which might create a novel opportunity for better antimicrobial stewardship in the future.

## Supplementary Material

Supplemental Material
