# Supplementary figures and images for: Genomic epidemiology and characterisation of penicillin-sensitive Staphylococcus aureus isolates from invasive bloodstream infections in China: an increasing prevalence and higher diversity in genetic typing be revealed
Source: Emerg Microbes Infect. 2022 Jan 21;11(1):326–36. doi: 10.1080/22221751.2022.2027218 (PMC8786255; doi:10.1080/22221751.2022.2027218)

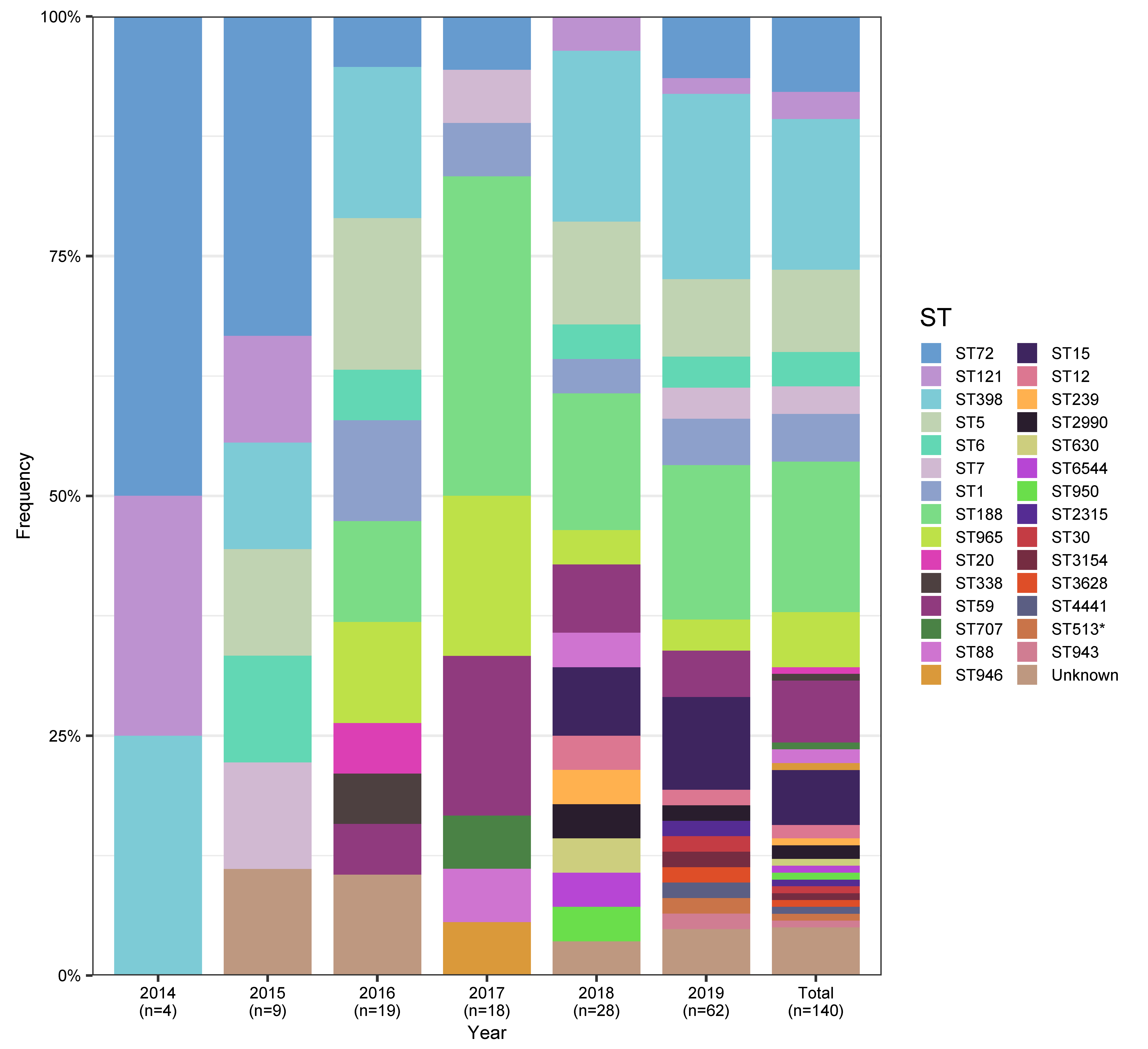

Supplement: Supplemental Material [file TEMI_A_2027218_SM6234.zip › 2027218_Suppl files/Figure S1.tiff]

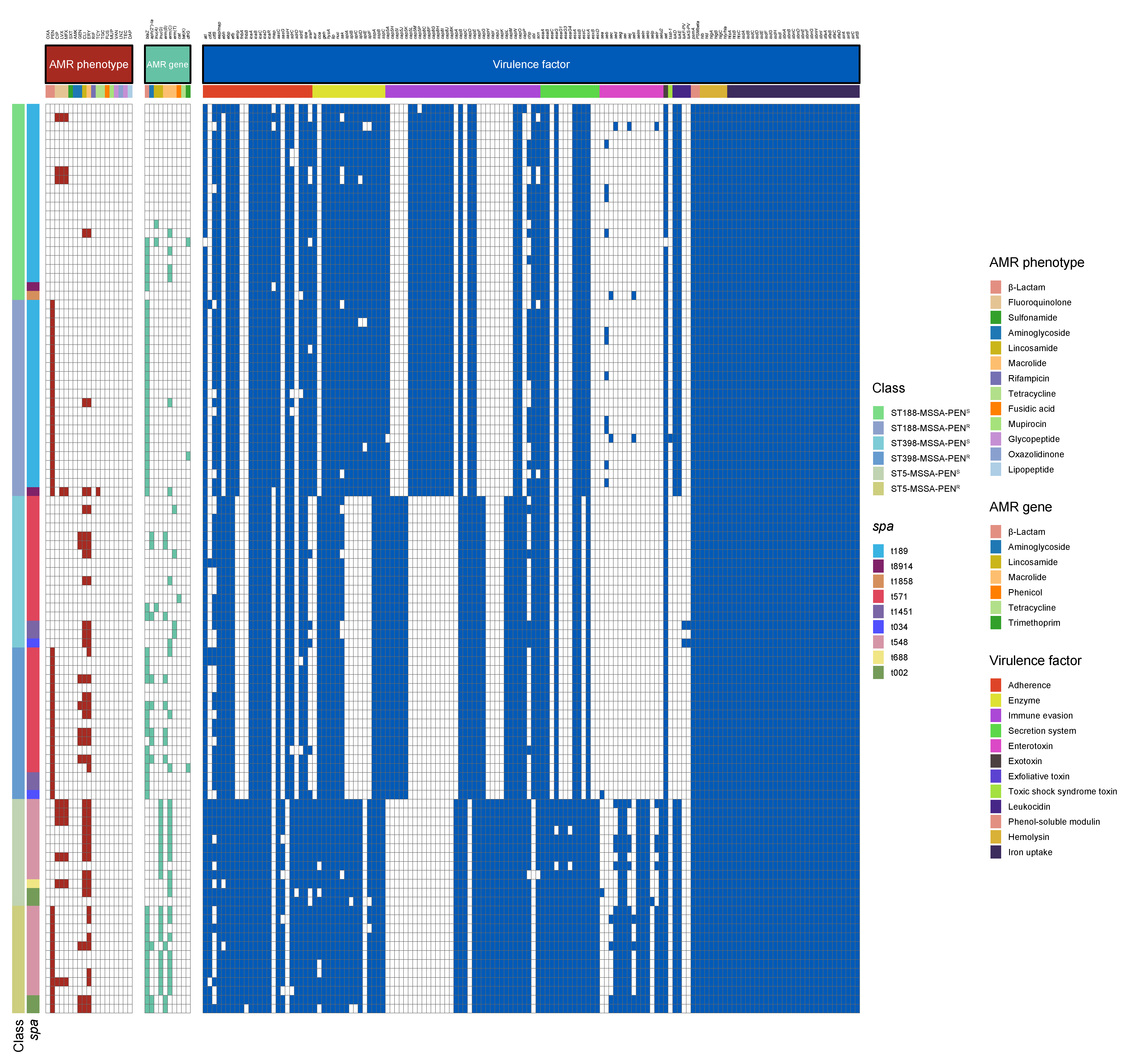

Supplement: Supplemental Material [file TEMI_A_2027218_SM6234.zip › 2027218_Suppl files/Figure S4.tiff]
